# Fascaplysin Sensitizes Anti-Cancer Effects of Drugs Targeting AKT and AMPK

**DOI:** 10.3390/molecules23010042

**Published:** 2017-12-24

**Authors:** Taek-In Oh, Jun Ho Lee, Seongman Kim, Taek-Jin Nam, Young-Seon Kim, Byeong Mo Kim, Woo Jong Yim, Ji-Hong Lim

**Affiliations:** 1 Department of Biomedical Chemistry, College of Biomedical & Health Science, Konkuk University, Chungju 27478, Chungbuk, Korea; dk1050@kku.ac.kr (T.-I.O.); seregay@kku.ac.kr (J.H.L.); 201017592@kku.ac.kr (S.K.); tj1994s@naver.com (T.-J.N.); yskim0801@kku.ac.kr (Y.-S.K.); 2 Severance Integrative Research Institute for Cerebral & Cardiovascular Diseases (SIRIC), Yonsei University College of Medicine, 50 Yonsei-ro, Seodaemun-gu, Seoul 03722, Korea; bkim2@yuhs.ac; 3 Jung-Ang Microbe Research Institute (JM), 398, Jikji-daero, Heungdeok-gu, Cheongju 28576, Chungbuk, Korea; ywj0808@naver.com; 4 Nanotechnology Research Center, Konkuk University, Chungju 27478, Korea

**Keywords:** fascaplysin, AKT, AMPK, chemoresistance, cancer

## Abstract

Fascaplysin, a natural product isolated from marine sponges, is a potential candidate for the development of anti-cancer drugs. However, the mechanism underlying its therapeutic effect of strengthening anti-cancer efficacy of other drugs is poorly understood. Here, we found that fascaplysin increases phosphorylation of protein kinase B (PKB), also known as AKT, and adenosine monophosphate-activated protein kinase (AMPK), which are considered therapeutic targets for cancer treatment due to their anti-apoptotic or pro-survival functions in cancer. A cell viability assay revealed that pharmacological suppression of AKT using LY294002 enhanced the anti-cancer effect of fascaplysin in various cancer cells. Similarly, fascaplysin was observed to have improved anti-cancer effects in combination with compound **C**, a selective AMPK inhibitor. Another challenge showed that fascaplysin increased the efficacy of methotrexate (MTX)-mediated cancer therapy by suppressing genes related to folate and purine metabolism. Overall, these results suggest that fascaplysin may be useful for improving the anti-cancer efficacy of targeted anti-cancer drugs, such as inhibitors of phosphoinositide 3-kinase AKT signaling, and chemotherapeutic agents, such as MTX.

## 1. Introduction

Chemoresistance acquired by genetic evolution during cancer treatment is closely linked to cancer recurrence and dissemination and patient death [1]. Overcoming chemoresistance is therefore an important challenge in the reduction of cancer-related deaths.

Fascaplysin is isolated from marine sponges and exerts anti-cancer effects, including the suppression of growth, angiogenesis, and metastasis in several cancer cells, through inhibition of cyclin-dependent kinase 4 (CDK4) [2,3,4,5]. Recently, we reported that fascaplysin exerts anti-cancer effects through the down-regulation of survivin and hypoxia inducible factor-1ɑ (HIF-1ɑ) by suppressing mechanistic target of rapamycin (mTOR)-eukaryotic translation initiation factor 4E-binding protein 1 (4EBP1)-mediated 5′-cap-dependent translation [5]. In addition, fascaplysin exerts anti-angiogenic effects by suppressing vascular endothelial growth factor receptor 2 (VEGFR2) through non-competitive inhibition of Asp-Phe-Gly (DFG)-out [6]. However, ways to improve the anti-cancer efficacy of fascaplysin for clinical application are poorly understood.

The activation of AKT signaling, which has pro-survival and anti-apoptotic functions, is commonly observed in several cancers [7,8]. It is also known that reactivation of AKT signaling confers resistance to chemotherapy using doxorubicin and etoposide [9]. Indeed, overexpression of AKT has shown that cancer cells are more highly resistant to paclitaxel in ovarian cancer [10]. Based on this background, it is becoming clear that selective inhibition of AKT signaling could improve the anti-cancer efficacy of chemotherapeutic agents. 

Adenosine monophosphate-activated protein kinase (AMPK), a major indicator of intracellular energy status, is phosphorylated and activated under nutrient deprivation for effective energy production [11,12]. AMPK-mediated autophagy in cancer could activate a cell survival mechanism that is activated by energy deprivation, chemotherapy, and hypoxia [13]. Paradoxically, activation of AMPK signaling often confers sensitivity to chemotherapeutic agents by promoting apoptosis [14]. Recently, a therapeutic approach has been described, by which pharmacological activation of AMPK signaling enhances the anti-cancer effects of methotrexate (MTX) by suppressing folate and purine metabolism [15,16]. AMPK signaling is therefore a potential target for decreasing cancer mortality. 

In the present study, we identified that fascaplysin unexpectedly increases AKT and AMPK phosphorylation. As activation of AKT and AMPK signaling causes resistance to anti-cancer therapy through its anti-apoptotic function, we hypothesized that the suppression of AKT and AMPK signaling may improve the anti-cancer efficacy of fascaplysin. Here, our results showed that LY294002, a selective inhibitor of PI3K-AKT signaling, and compound **C**, an AMPK inhibitor, dramatically reduce cancer cell growth when used in combination with fascaplysin. In addition, we found that fascaplysin is sufficient to enhance the anti-cancer efficacy of MTX.

## 2. Results

### 2.1. Fascaplysin Increases AKT Phosphorylation and DNA Damage Signaling 

**Figure 1:**
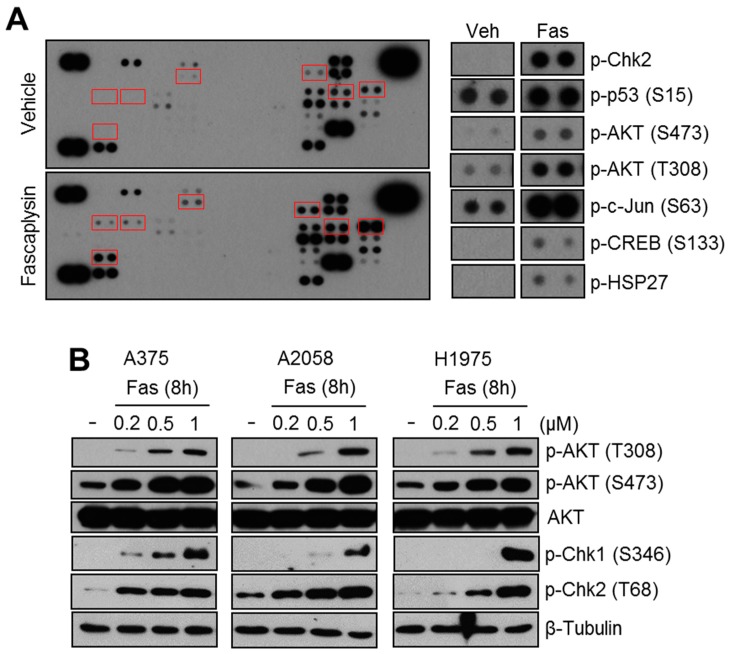
Fascaplysin increases AKT phosphorylation and DNA damage-induced signaling. (**A**) A375 melanoma cells were incubated with fascaplysin or dimethyl sulfoxide (DMSO) control for 8 h, and 300 μg of total cell lysate was reacted with a phosphorylated kinase proteome profiler membrane. Up- or down-regulated kinases were identified. (**B**) A375, A2058, and H1975 cells were cultured in the absence or presence of the indicated concentrations of fascaplysin for 8 h, and then proteins were analyzed by Western blotting.

The reactivation of anti-apoptotic or pro-survival signaling during cancer treatment causes poor anti-cancer efficacy, due to chemoresistance [1]. To develop a strategy to improve the anti-cancer efficacy of fascaplysin, we investigated the activation of anti-apoptotic or pro-survival signaling upon fascaplysin treatment using a phosphorylated kinase proteome array. Unexpectedly, increased AKT phosphorylation and DNA damage signaling molecules, such as phospho-Chk2 and phospho-p53, were observed in fascaplysin-treated A375 melanoma cells (Figure 1A). To validate whether fascaplysin increases AKT, Chk1, and Chk2 phosphorylation, we measured phosphorylation status them in response to fascaplysin in A375, A2058, and H1975 which have shown suppressive effect of fascaplysin on 4EBP1 and p70S6K1 phosphorylation in previous report [6]. Figure 1B shows that fascaplysin strongly increased the phosphorylation of AKT, Chk1, and Chk2. These results suggest that AKT phosphorylation could confer resistance to fascaplysin-induced cancer cell death. 

### 2.2. Suppression of AKT Activation Synergizes the Anti-Cancer Effects of Fascaplysin 

**Figure 2:**
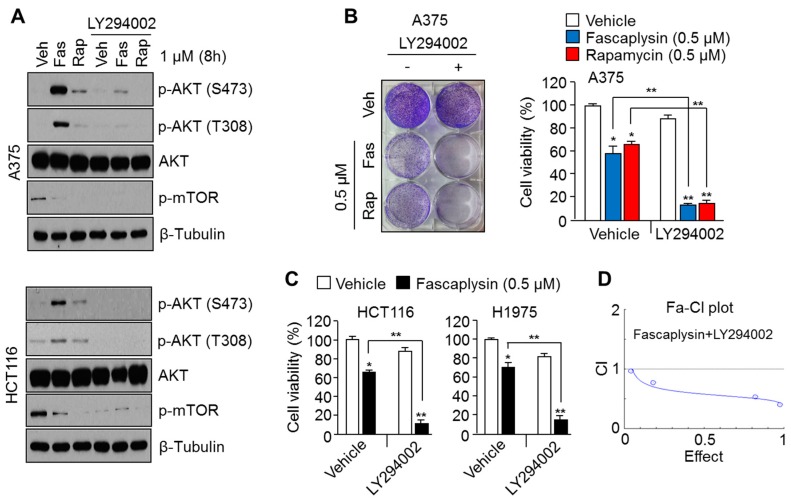
Pharmacological targeting of fascaplysin-induced AKT phosphorylation enhances the anti-cancer effect of fascaplysin. (**A**) A375 and HCT116 cells were pre-incubated with 10 μM of LY294002 for 20 min, and were then further treated with 1 μM of fascaplysin or rapamycin for 8 h. Protein levels were analyzed by Western blotting. (**B**,**C**) A375 cells were pre-incubated with 10 μM of LY294002 for 20 min, and were then treated with 0.5 μM of fascaplysin or rapamycin for 36 h. The cell viability upon treatment of fascaplysin and LY294002 were measured in HCT116 and H1975 cells. Cell viability was measured by using the crystal violet staining assay described in Methods and Materials. (**D**) A375 cells were incubated with series of dose of fascaplysin and LY294002 for 48 h, and then cell viability was determined by crystal violet assay. The combination index of the synergistic effect of fascaplysin with LY294002 in A375 cells was calculated by the CalcuSyn software 2.1. Values are represented as the mean ± standard deviation (SD) of three independent experiments performed in triplicate; * *p* < 0.05 and ** *p* < 0.01.

AKT has been considered a therapeutic target for overcoming chemoresistance, due to its anti-apoptotic and pro-survival functions [8]. Indeed, the pharmacological suppression of AKT reactivation by rapamycin, an allosteric mTOR inhibitor, has been shown to improve the anti-cancer efficacy of rapamycin [17]. We therefore further examined whether pharmacological AKT inhibition using LY294002 enhances fascaplysin-induced cancer cell death. Figure 2A shows that the increased AKT phosphorylation upon fascaplysin or rapamycin treatment was significantly diminished by LY294002, a selective inhibitor of phosphoinositide 3-kinase (PI3K). Moreover, low concentrations of fascaplysin (0.5 μM) or rapamycin (0.5 μM) were sufficient to synergistically reduced cell viability approximately 50% in combination with LY294002, in A375 melanoma cells (Figure 2B). Consistently, increased anti-cancer effect upon combination with fascaplysin and LY294002 were observed in colorectal cancer (HCT116) and lung cancer (H1975) cell lines (Figure 2C). To show synergism of anti-cancer effect upon combination with fascaplysin and LY294002, we calculated the combination index with series of concentration of fascaplysin (0.2, 0.5, 1 and 2 μM) and LY294002 (0.2, 0.5, 1 and 2 μM) in A375 melanoma cells (Figure 2D) using CalcuSyn software 2.1 [18]. These results suggest that the pharmacological suppression of AKT signaling, which is reactivated upon fascaplysin treatment, may improve the anti-cancer efficacy of fascaplysin. 

### 2.3. Fascaplysin Causes Metabolic Stress and Increases AMPK Signaling 

**Figure 3:**
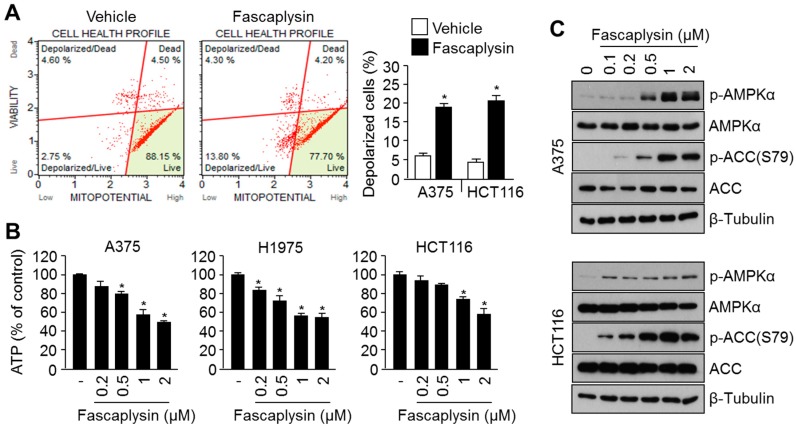
Fascaplysin causes mitochondrial depolarization and ATP depletion, resulting in increased AMPK phosphorylation and activation. (**A**) A375 and HCT116 cells were incubated with 1 μM of fascaplysin or DMSO control for 8 h, and then cells were used to analyze mitochondrial depolarization. Mitochondrial potential was measured using a Muse™ Mitopotential assay kit and a Mini Flow Cytometry Muse™ Cell Analyzer, as described in Methods and Materials. Values are represented as the mean ± standard deviation (SD) of two independent experiments performed in triplicate; * *p* < 0.05; (**B**) A375, H1975, and HCT116 cells were cultured with various concentrations of fascaplysin or DMSO control for 8 h, and then cellular ATP levels were measured using an ATP assay kit as described in Methods and Materials. Relative ATP levels were normalized using total protein concentrations and compared to that of the DMSO control. Values are represented as the mean ± standard deviation (SD) of two independent experiments performed in triplicate; * *p* < 0.05; (**C**) A375 or HCT116 cells were treated with the indicated concentrations of fascaplysin or DMSO for 8 h, and then protein levels were measured by Western blotting.

We previously reported that fascaplysin induces apoptosis through activation of caspases cascade [6], which is closely linked to mitochondrial stress [19]. We therefore examined whether fascaplysin causes mitochondrial damage and metabolic stress. Figure 3A shows that fascaplysin increased mitochondrial depolarization. In addition, severe ATP depletion was observed upon fascaplysin treatment in A375, HCT116, and H1975 cancer cells (Figure 3B). As AMPK signaling is activated upon metabolic stress to prevent cell death [11,12], activation of AMPK was measured upon fascaplysin treatment. Figure 3C shows that fascaplysin dramatically increased phosphorylation of AMPK and its target kinase, acetyl-coA carboxylase (ACC). These results reveal that fascaplysin increases the activation of AMPK signaling, which may be associated with chemoresistance and adaptation to metabolic stress. 

### 2.4. Suppression of Fascaplysin-Induced AMPK Activation Synergizes Anti-Cancer Efficacy of Fascaplysin 

**Figure 4:**
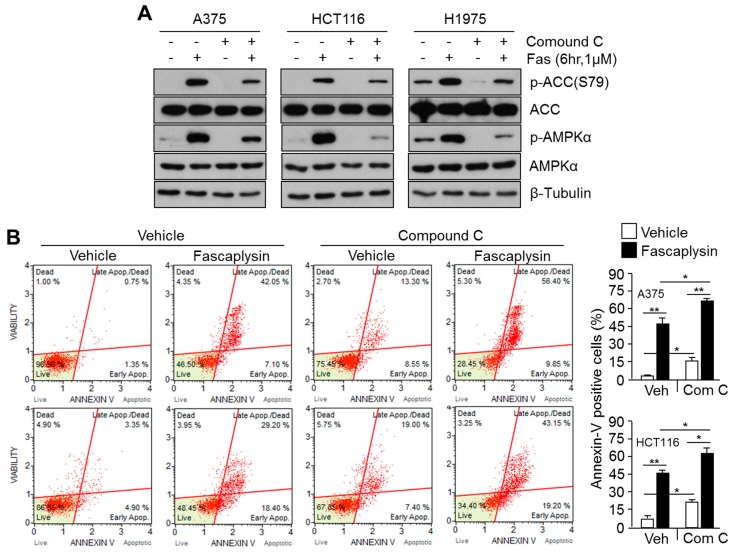
Suppression of fascaplysin-induced AMPK activation enhances the anti-cancer effect of fascaplysin. (**A**) A375, HCT116, and H1975 cells were pre-incubated with 5 μM of compound **C** for 10 min, and were then further treated with 1 μM of fascaplysin for 6 h. The protein levels were measured by Western blotting; (**B**) Compound **C** (5 μM) pre-treated A375 or HCT116 cells were further incubated with fascaplysin (1 μM) for 24 h, and then apoptotic cell numbers were measured using a Muse™ Annexin-V assay kit and a Mini Flow Cytometry Muse™ Cell Analyzer. Values are represented as the mean ± standard deviation (SD) of three independent experiments performed in duplicate; * *p* < 0.05 and ** *p* < 0.01.

AMPK signaling has been also considered as a therapeutic target for overcoming resistance to anti-cancer drugs during cancer treatment [20,21]. Here, we examined whether targeting AMPK activation synergistically induces apoptosis in fascaplysin-treated cancer cells. Figure 4A shows that compound **C** was sufficient to block AMPK phosphorylation and activation upon fascaplysin treatment, as well as ACC phosphorylation. Moreover, compound **C** notably increased fascaplysin-induced apoptosis in A375 and HCT116 cancer cells (Figure 4B), suggesting that the pharmacological suppression of fascaplysin-induced AMPK activation using a selective AMPK inhibitor, compound **C**, synergistically increases apoptosis of cancer cells.

### 2.5. MTX Enhances the Anti-Cancer Efficacy of Fascaplysin 

**Figure 5:**
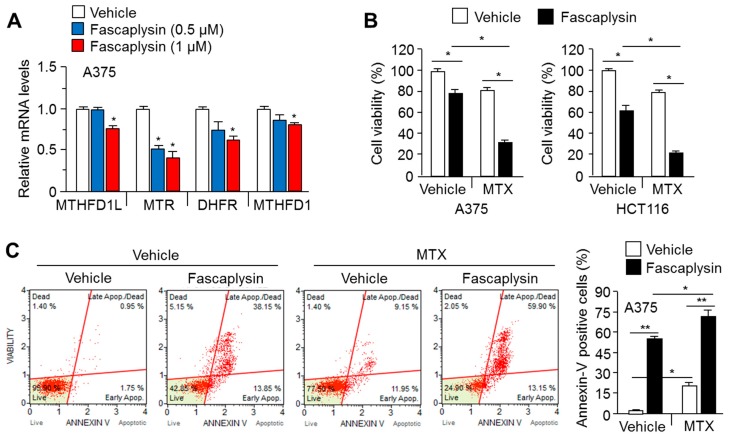
Anti-folate cancer therapy by methotrexate (MTX) enhances the anti-cancer effect of fascaplysin. (**A**) A375 cells were incubated with the indicated concentrations of fascaplysin or DMSO control for 24 h, and then mRNA levels were measured using quantitative real time (RT)-PCR. Values are represented as the mean ± standard deviation (SD) of three independent experiments performed in duplicate; * *p* < 0.05; (**B**) A375 and HCT116 cells were pre-incubated with 0.1 μM of MTX for 10 min, and were then further treated with 0.5 μM of fascaplysin or DMSO control for 24 h. Cell viability was measured using a crystal violet staining assay. Values are represented as the mean ± standard deviation (SD) of three independent experiments performed in triplicate; * *p* < 0.05; (**C**) A375 cells were pre-treated with 0.1 μM of MTX for 10 min, and were then further incubated with fascaplysin (0.5 μM) or DMSO control for 24 h. The apoptotic cell numbers were then measured: values are represented as the mean ± standard deviation (SD) of three independent experiments performed in duplicate; * *p* < 0.05 and ** *p* < 0.01.

Previous reports have shown that the activation of AMPK signaling using 5-aminoimidazole-4-carboxamide ribonucleotide, known as AICAR, can improve MTX-based cancer therapy via the suppression of folate and purine metabolism [15,16]. Here, we further investigated whether fascaplysin suppresses the expression of genes related to folate and purine metabolism. Figure 5A shows that fascaplysin dramatically decreased the expression of such genes, including methylenetetrahydrofolate dehydrogenase (NADP^+^-dependent) 1-like (*MTHFD1L*), 5-methyltetrahydrofolate-homocysteine methyltransferase (*MTR*), dihydrofolate reductase (*DHFR*) and methylenetetrahydrofolate dehydrogenase 1 (MTHFD1). As folate and purine metabolism are essential biological processes that supply nucleotides as building blocks for cell proliferation, cancer cell viability upon combination treatment with fascaplysin and MTX was measured. Figure 5B shows that MTX synergistically decreased cell viability by approximately 40–50% upon fascaplysin treatment in A375 and HCT116 cells. Consistent with this, fascaplysin-induced cancer cell was significantly increased approximately 20% by the addition of MTX (Figure 5C). These results indicate that fascaplysin suppresses folate and purine metabolism, and that MTX is sufficient to improve the anti-cancer efficacy of fascaplysin. 

## 3. Discussion

Fascaplysin, as a natural compound isolated from marine sponge, exhibits anti-cancer effects in multiple types of cancer cell, including small cell lung cancer [22], melanoma [23], and glioma [24] through suppression of CDK4-mediated cell cycle progression. However, it is still poorly understood whether fascaplysin could improve anti-cancer effects of chemotherapeutic drugs. In the present study, pro-survival or anti-apoptotic factors such as AKT and AMPK unexpectedly increased in fascaplysin-treated cancer cells. We also uncovered that fascaplysin sensitized melanoma, colorectal, and lung cancer cells to the apoptotic effects of chemotherapeutic agents or targeted anti-cancer drugs. Indeed, the reactivation of AKT was observed upon fascaplysin treatment. However, previous reports showed that fascaplysin decreases AKT phosphorylation in HL-60 leukemia cells [25]. Rapamycin, a well-established selective mTOR inhibitor, reactivates AKT via mammalian target of rapamycin complex 2 (mTORC2) signaling in several lung cancer cell lines, including A549, H358, and H460 but not HL-60 leukemia cells [26,27,28]. Fascaplysin is therefore believed to have similar effects as rapamycin, and the AKT reactivation upon fascaplysin treatment might be dependent on cellular context. Nevertheless, the precise molecular mechanism by which fascaplysin reactivates AKT should be further investigated. As hyperactivation of AKT signaling causes drug resistance during cancer treatment [8], we investigated and found that combining fascaplysin with LY294002 largely reduced viability of A375 (melanoma), HCT116 (colorectal cancer), and H1975 (lung cancer) cells. As acquired resistance to LY294002, a targeted anti-cancer drug, has also been reported in several cancers [1], fascaplysin may be useful in improving the anti-cancer efficacy of PI3K-AKT-targeting drugs.

The intercalation of fascaplysin into double-stranded calf thymus DNA has been observed [29]. Moreover, a previous report showed that the non-planar tryptoline analog of fascaplysin, *N*-(biphenyl-2-yl) tryptoline (BPT, 6), attenuates cancer cell growth by increasing p53 in vitro and in vivo [30]. Consistent with these previous works, a marked increase in Chk2 and p53 phosphorylation (which are closely linked to DNA damage) was observed in fascaplysin-treated cells. 

Reduced intracellular ATP levels and mitochondrial membrane potential were observed in A375, H1975, and HCT116 cancer cells. However, a previous report showed that fascaplysin, as a cyclin-dependent kinase 4 (CDK4) inhibitor, increases the activity of PPARγ-coactivator-1α (PGC1α). This occurs via the suppression of GCN5-mediated acetylation, without any cytotoxicity in non-proliferative primary hepatocytes [31]. PGC1α is a master transcriptional coactivator for mitochondrial biogenesis and the maintenance of energy balance in several metabolic tissues [32]. However, our findings show that fascaplysin causes metabolic stress, with decreased mitochondrial membrane potential and intracellular ATP levels. Thus, we speculate that fascaplysin could selectively kill cancer cells but not normal differentiated cells, through induction of metabolic stress and apoptosis. Consistent with ATP depletion, AMPK phosphorylation was observed in fascaplysin-treated cancer cells. AMPK activation under metabolic stress helps overcome the stress [11,12]. In addition, AMPK-mediated autophagy confers resistance to several anti-cancer drugs [13,20,21]. It was therefore thought that AMPK activation may confer resistance to fascaplysin-induced apoptosis through autophagy and metabolic compensation. In this study, we found that pharmacological inhibition of AMPK using compound **C** synergistically enhanced fascaplysin-induced apoptosis in A375 and HCT116 cancer cells. Thus, our results suggest that the combination of fascaplysin with AMPK inhibitor may effectively suppress cancer growth. 

One-carbon-based folate and purine metabolism supports building blocks for maintaining intracellular nucleotides [33]. MTX, a potent inhibitor of dihydrofolate reductase (*DHFR*), an essential enzyme for folate metabolism, functionally reduces the biosynthesis of purines and consequently DNA synthesis [33]. Although MTX is an effective therapeutic agent for osteosarcoma and breast cancer, drug resistance with increased *DHFR* gene expression has been observed in several cancers [34]. It has recently been reported that the suppression of folate and purine metabolism by AMPK activation synergistically increases the anti-cancer efficacy of MTX [15]. In this study, we found that fascaplysin suppressed the expression of genes related to folate and purine metabolism, such as *MTR* and *DHFR*. Importantly, fascaplysin sensitized cancer cells to the apoptotic effect of MTX. Notably, our results suggest that fascaplysin has potential as a drug to overcome resistance to MTX-based cancer treatment.

Taken together, our results detail the several beneficial effects of fascaplysin, which allow it to overcome drug resistance for cancer treatment: (1) fascaplysin sensitizes cells to the anti-cancer effect of AKT inhibition-based cancer treatment; (2) fascaplysin causes metabolic stress with decreased intracellular ATP levels and synergistically increases cancer cell apoptosis upon AMPK inhibitor treatment; and (3) fascaplysin suppresses the expression of several folate and purine metabolism-related genes, such as *MTR* and *DHFR*, and sensitizes cells to apoptosis caused by MTX.

## 4. Materials and Methods

### 4.1. Reagents and Antibodies

Fascaplysin, Compound **C**, and MTX were purchased from Selleck Chemicals (Houston, TX, USA). Rapamycin and LY294002 were purchased from Santa Cruz Biotechnology (Dallas, TX, USA). For Western blotting, antibodies against phospho-AMPKα (CST-2535), total-AMPKα (CST-5831), phospho-AKT-S473 (CST-4060), phospho-AKT-T308 (CST-13038), total-AKT (CST-4691), phospho-ACC (CST-11818), total-ACC (CST-3676), and β-tubulin (sc-9104) were purchased from Cell Signaling Technology (Danvers, MA, USA) and Santa Cruz Biotechnology (Dallas, TX, USA). 

### 4.2. Cell Culture and Cell Viability Assay

Human melanoma (A375 and A2058), lung cancer (H1975), and colorectal cancer (HCT116) cells were maintained in Dulbecco’s modified Eagle’s medium (DMEM) (Thermo Fisher Scientific, Waltham, MA, USA) supplemented with fetal bovine serum (10%) (Thermo Fisher Scientific, Waltham, MA, USA). Cell viability was measured by crystal violet staining and analysis [5]. The incubated cells for the various experimental conditions were fixed using formaldehyde and stained with 0.5% crystal violet staining solution (Sigma Aldrich, St. Louis, MO, USA). To evaluate cell viability, crystal violet-stained cells were solubilized using lysis buffer containing 1% sodium dodecyl sulfate (SDS), and then, optical density was measured at 570 nm (OD570) using an absorbance reader (BioTek, Winooski, VT, USA). 

### 4.3. Western Blotting

Total protein was extracted using lysis buffer (1% IGEPAL, 150 mM NaCl, 50 mM Tris-HCl (pH 7.9), 10 mM NaF, 0.1 mM EDTA, and protease inhibitor cocktail (Sigma Aldrich, St. Louis, MO, USA), and then mixed with sample buffer containing SDS prior to electrophoresis [6]. Total protein was subjected to sodium dodecyl sulfate-polyacrylamide gel electrophoresis (SDS-PAGE) and then transferred to a PVDF membrane (EMD Millipore, Burlington, MA, USA). The transferred membranes were incubated with primary antibodies (1:1000–10,000) at 4 °C overnight, and were then incubated with secondary antibodies (1:10,000) for 1 h at room temperature. Protein signals were detected using an enhanced chemiluminescence (ECL) Prime kit (GE healthcare, Pittsburgh, PA, USA). 

### 4.4. Quantitative Real-Time PCR

Two micrograms of total RNA were isolated using TRIzol (Thermo Fisher Scientific, Waltham, MA, USA) for cDNA synthesis [6]. The mRNA expression levels were measured using quantitative real-time PCR with cDNA and SYBR Green PCR Master mixture (Thermo Fisher Scientific, Waltham, MA, USA). The PCR primer sequences (5′-3′) were as follows: AGTCCTGGCATGGTGCTC and TGTGCCAAGGGACTTCATCT for MTHFD1L; TTCACAAGCAGATGGTAGGC and GCTATGGTGGTCATGGCTTT for MTR; AAATGAGCTCCTTGTGGAGG and ACCTGGTTCTCCATTCCTGA for DHFR; and TTGATTTTTCAGTCTCGCCC and TATTGGTGGTGTCCATCGTG for MTHFD1.

### 4.5. Proteome Profiler Array

Before the phosphorylated-proteome analysis, 1 × 10^7^ cells/ml were harvested and rinsed using cold phosphate-buffered saline (PBS) (Sigma Aldrich, St. Louis, MO, USA), and then solubilized in lysis buffer from a Human Phospho-Kinase Array Kit (R&D systems, Minneapolis, MN, USA). The provided nitrocellulose membranes, conjugated to biotinylated antibodies against multiple types of phospho-kinase proteins, were incubated with 300 μg of cell lysate, overnight at 4 °C. After the primary antibody reaction, the samples were washed using 20 mL of 1× wash buffer for 10 min at room temperature, and then the membranes were incubated with streptavidin-HRP (1:10,000) in 1× array buffer for 30 min at room temperature. Protein signals were then detected using a ChemiReagent Mixture.

### 4.6. Apoptosis Assays

Apoptosis assays were performed using Muse™ Annexin V & Dead Cell kit reagent (EMD Millipore, Burlington, MA, USA). Cells were cultured at a density of 1 × 10^5^ cells in six-well cell culture plates. After stabilization, cells were incubated for 24 h with fascaplysin, compound **C**, or MTX. Cells were washed with cold PBS, and then adherent cells were collected into a fresh tube using trypsin-EDTA (0.25%). Cell pellets were resuspended in 1 mL of fresh medium, and then the resuspended cells (100 µL) were incubated for 20 min with Muse™ Annexin V & Dead Cell kit reagent. After the reaction, all samples were analyzed using a Mini Flow Cytometry Muse™ Cell Analyzer (EMD Millipore, Burlington, MA, USA).

### 4.7. Mitochondrial Membrane Potential Assay

Mitopotential Assays were performed using a Muse^®^ Mitopotential assay kit. Cells were cultured at 1 × 10^5^ cells per well in six-well cell culture plates. They were treated with fascaplysin (1 μM) for 24 h, and then were washed with cold PBS. Adherent cells were collected in a fresh tube, and the cell pellet was then washed and resuspended in 1× assay buffer from the assay kit. The resuspended cells (100 µL) were incubated with 95 μL of Mitopotential working solution and 5 μL of 7-AAD reagent. Mixed samples were incubated for 20 min in an incubator maintained at 37 °C. After the reaction, mitochondrial potential and cell numbers were analyzed using a Mini Flow Cytometry Muse™ Cell Analyzer (EMD Millipore, Burlington, MA, USA).

### 4.8. ATP Assay

To measure intracellular ATP levels, 1 × 10^5^ cells were seeded in six-well cell culture plates. After stabilization for 24 h, cells were incubated with a range of concentrations of fascaplysin for 8 h. Cells were harvested and solubilized in 100 μL ATP assay buffer from an ATP Colorimetric/Fluorometric assay kit (Biovision, CA, USA). The solubilized samples were transferred into 96-well plates, and then mixed with 50 μL of ATP reaction solution, including an ATP probe and a developer. After incubation for 30 min at room temperature with protection from light, sample absorbance was measured at 570 nm using an absorbance reader (BioTek, Winooski, VT, USA).

### 4.9. Statistical Analysis

An unpaired Student’s *t*-test for two experimental comparisons and one-way ANOVA with Tukey post-test for multiple comparisons were used for data analysis. Data are represented as mean ± standard deviation (SD) values, which were considered statistically significant when *p* < 0.05.
